# Magnetic Resonance Imaging and Spectroscopy Assessment of Lower Extremity Skeletal Muscles in Boys with Duchenne Muscular Dystrophy: A Multicenter Cross Sectional Study

**DOI:** 10.1371/journal.pone.0106435

**Published:** 2014-09-09

**Authors:** Sean C. Forbes, Rebecca J. Willcocks, William T. Triplett, William D. Rooney, Donovan J. Lott, Dah-Jyuu Wang, Jim Pollaro, Claudia R. Senesac, Michael J. Daniels, Richard S. Finkel, Barry S. Russman, Barry J. Byrne, Erika L. Finanger, Gihan I. Tennekoon, Glenn A. Walter, H. Lee Sweeney, Krista Vandenborne

**Affiliations:** 1 Department of Physical Therapy, University of Florida, Gainesville, FL, United States of America; 2 Oregon Health & Science University, Portland, OR, United States of America; 3 The Children’s Hospital of Philadelphia, Philadelphia, PA, United States of America; 4 Department of Statistics & Data Sciences and Department of Integrative Biology, the University of Texas at Austin, Austin, TX, United States of America; 5 Nemours Children’s Hospital, Orlando, Florida, United States of America; 6 Department of Pediatrics and Molecular Genetics & Microbiology, Powell Gene Therapy Center, University of Florida, Gainesville, FL, United States of America; 7 Department of Physiology and Functional Genomics, University of Florida, Gainesville, FL, United States of America; 8 Department of Physiology, University of Pennsylvania, Philadelphia, PA, United States of America; National University of Singapore, Singapore

## Abstract

**Introduction:**

Duchenne muscular dystrophy (DMD) is an X-linked recessive disorder that results in functional deficits. However, these functional declines are often not able to be quantified in clinical trials for DMD until after age 7. In this study, we hypothesized that ^1^H_2_O T_2_ derived using ^1^H-MRS and MRI-T_2_ will be sensitive to muscle involvement at a young age (5–7 years) consistent with increased inflammation and muscle damage in a large cohort of DMD subjects compared to controls.

**Methods:**

MR data were acquired from 123 boys with DMD (ages 5–14 years; mean 8.6 SD 2.2 years) and 31 healthy controls (age 9.7 SD 2.3 years) using 3-Tesla MRI instruments at three institutions (University of Florida, Oregon Health & Science University, and Children’s Hospital of Philadelphia). T_2_-weighted multi-slice spin echo (SE) axial images and single voxel ^1^H-MRS were acquired from the lower leg and thigh to measure lipid fraction and ^1^H_2_O T_2_.

**Results:**

MRI-T_2_, ^1^H_2_O T_2_, and lipid fraction were greater (p<0.05) in DMD compared to controls. In the youngest age group, DMD values were different (p<0.05) than controls for the soleus MRI-T_2_, ^1^H_2_O T_2_ and lipid fraction and vastus lateralis MRI-T_2_ and ^1^H_2_O T_2_. In the boys with DMD, MRI-T_2_ and lipid fraction were greater (p<0.05) in the oldest age group (11–14 years) than the youngest age group (5–6.9 years), while ^1^H_2_O T_2_ was lower in the oldest age group compared to the young age group.

**Discussion:**

Overall, MR measures of T_2_ and lipid fraction revealed differences between DMD and Controls. Furthermore, MRI-T_2_ was greater in the older age group compared to the young age group, which was associated with higher lipid fractions. Overall, MR measures of T_2_ and lipid fraction show excellent sensitivity to DMD disease pathologies and potential therapeutic interventions in DMD, even in the younger boys.

## Introduction

Duchenne muscular dystrophy (DMD) is an X-linked recessive disorder caused by mutations in the dystrophin gene [1]. DMD has an incidence of 1 in 3,600–6,000 male births and is characterized by progressive muscle deterioration, loss of functional abilities, and reduced life expectancy [2]. Symptoms of DMD are usually recognized between two and three years of age, and include delayed walking and running, and difficulty climbing stairs [2]. Boys with DMD are typically diagnosed by five years of age [3], [4].

Currently there is no cure for DMD, although there are a number of therapeutic interventions that have shown promise in preclinical and early clinical trials, including viral delivery of microdystrophin genes [5], exon skipping [6], and small molecule therapies, such as ataluren [7]. Interventions for boys with DMD may be most effective in young boys with DMD who have not yet experienced significant muscle deterioration and atrophy [8]. Therefore, there is a need for sensitive biomarkers to measure muscle involvement to evaluate the effects of potential therapeutic interventions in young boys with DMD.

The six minute walk test (6MWT) has been utilized as the primary outcome measure in two recent phase IIb/III clinical trials in DMD [9]. However, functional decline in motor performance, such as reduced distance walked in the 6MWT, is often not observed in DMD until after age seven [10]. Furthermore, the 6MWT has been criticized for its dependence on subject attention span, motivation, and neuromuscular coordination [9]. In addition to the 6MWT, comprehensive motor evaluations have been proposed using a battery of functional tests, such as the North Star Ambulatory Assessment, which uses a composite score of 17 items [11], [12]. This score was recently shown to be sensitive to detect differences between corticosteroid regimens in DMD [13].

Magnetic resonance imaging (MRI) and spectroscopy (MRS) may also have the potential to be sensitive markers of muscle involvement in DMD. The MRI transverse relaxation time constant (MRI-T_2_) that represents the overall bulk T_2_ of the region of interest, and is influenced by both lipid and ^1^H_2_O components [14], [15], has previously been observed to differentiate boys with DMD from controls [16]. Furthermore, lipid infiltration has been associated with disease progression, age, and clinical functional tests in DMD as assessed using MRI 3-point Dixon [17], [18] and single voxel ^1^H-MRS [19], [20]. Also, ^1^H_2_O T_2_ of skeletal muscles assessed using single voxel ^1^H-MRS, a measure independent of lipid infiltration, has been shown to be greater in DMD compared to controls [21], [22], indicating elevated muscle damage and inflammation/edema [23]. As a result, lipid fraction, ^1^H_2_O T_2_, and MRI-T_2_ all have the potential to be effective in monitoring disease progression in DMD. In a previous study, we have shown that using standardized procedures these quantitative MR measures of muscle composition can be reproducibly implemented within and across multiple sites [21].

Therefore, using a multicenter trial design with a large cohort of ambulatory boys with DMD and controls of different age groups (5–6.9, 7–8.9, 9–10.9, and 11–14 years) we measured lipid fraction, ^1^H_2_O T_2_, and MRI-T_2_ of muscles in the lower and upper leg to assess the potential of these measures to monitor muscle involvement in DMD and ultimately test potential therapeutic interventions in future studies. We hypothesized that: 1) ^1^H_2_O T_2_ is sensitive to muscle disease, even at a young age (5–6.9 years), consistent with increased muscle damage and inflammation/edema in DMD compared to controls; 2) ^1^H_2_O T_2_ and MRI-T_2_ will be altered with age and disease progression; and 3) MRI-T_2_ will be associated with lipid fraction and both MRI-T_2_ and lipid fraction will increase with age groups in lower and upper leg muscles of DMD.

## Methods

The data from this study were collected as a part of the multi-center Imaging DMD study (http://imagingdmd.org/) ([21], [22], [24]). The study was approved by the Institutional Review Boards (IRB) at the University of Florida (UF), Oregon Health & Science University (OHSU), and Children’s Hospital of Philadelphia (CHOP). The study was in compliance with the Health Insurance Portability and Accountability Act (HIPAA) and an informed written assent/consent was obtained from the subject/guardian prior to participation in the study.

### Participants

MR data were acquired from 123 boys with DMD (ages 5–14 years; mean 8.6 SD 2.2 years) and 31 healthy controls (ages 5–14 years; age 9.7 SD 2.3). The boys with DMD were confirmed by genetic testing, ambulatory, and 86 were receiving corticosteroids. The controls were similar in age to the boys with DMD. All participants were asked to avoid any excessive physical activity beyond their normal levels for three days prior to the study.

### MR acquisition

MR data were acquired from the lower leg and thigh from three centers by using a 3T Achieva Quasar Dual MRI instrument (Philips, Best, the Netherlands), a 3T Magnetom TIM Trio MRI instrument (Siemens, Erlangen, Germany), or a 3T Magnetom Verio MRI instrument (Siemens), as previously described [21]. Radiofrequency coil configurations differed between centers. For the lower leg, a transmit-receive quadrature extremity coil or an eight-channel sensitivity encoding volume receive-only knee coil was used. For the thigh, a two-channel surface coil, a body matrix array coil, or a transmit-receive quadrature extremity coil was used.

**Figure 1:**
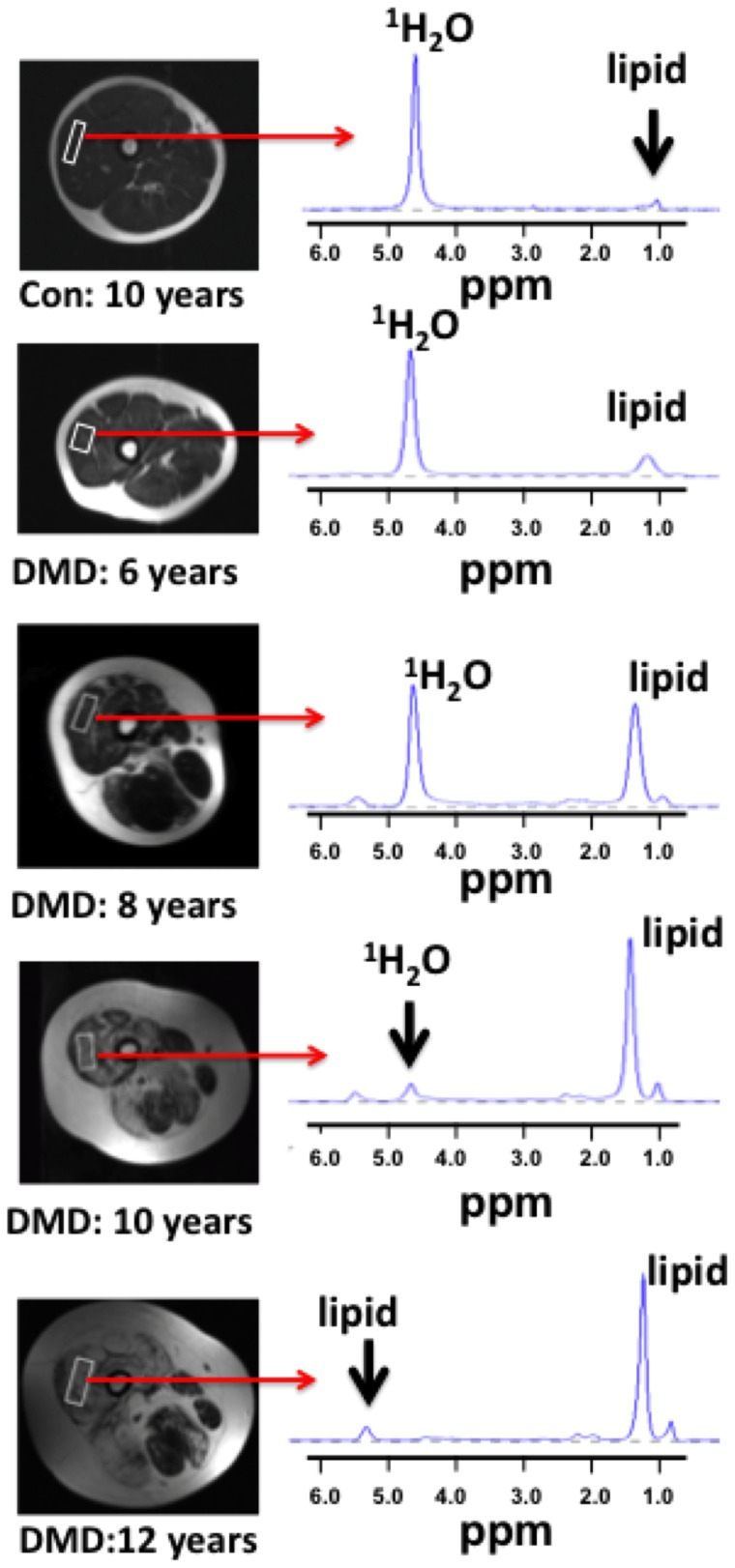
Example upper leg axial spin echo (SE) images (TE 60 ms) with single voxel ^1^H-MRS spectra (TE 108 ms) from the vastus lateralis of a control and boys with DMD at different ages.

Subjects lay supine in the bore of the magnet with the leg secured using foam padding and rice bags. MRI/MRS measurements were collected from the upper and lower regions of the right leg. T_2_-weighted multi-slice spin echo (SE) axial images were acquired (4–6 slices, 7 mm slices, 3.5 mm gap; 16 TE’s, 20–320 ms evenly spaced; TR = 3 s) (Fig. 1). The refocusing pulse width was set at 1.5 times the excitation pulse width on all scanners. Single voxel ^1^H-MRS data were acquired (TE = 108 ms; TR = 3 s; NA = 64) for assessment of lipid fraction using stimulated-echo acquisition mode (STEAM) from the soleus (Sol) and vastus lateralis (VL; Fig. 1) [25], [26]. Finally, ^1^H spectroscopic relaxometry was performed using STEAM in the Sol (16 TE’s non-linearly spaced from 11–288 ms; TR = 9 s; NA = 4) and VL (4 TE’s non-linearly spaced from 11–243 ms; TR = 9 s; NA = 4). The total acquisition time including subject set-up was approximately 75 minutes.

### MR Analysis

The MR data were transferred to and analyzed at a single center. Imaging-based T_2_ values were measured for the tibialis anterior (TA), tibialis posterior (TP), peroneus longus and brevis (Per), soleus (Sol), medial gastrocnemius (MG), biceps femoris long head (BFLH), vastus lateralis (VL), and gracilis (Gra). Each of these measures used an average of 3 contiguous axial slices. T_2_ maps were calculated for lower leg and thigh muscles by voxel-wise estimation of T_2_ by fitting a single exponential equation to the magnitude signal intensity (S) from spin-echo images with TE values from 40–100 ms; S(TE) = S_0_*exp(-TE/T_2_) by varying S_0_ and T_2_ [16], [21]. The initial TE was not included to minimize the effect of stimulated echoes on the calculation of T_2_ [27]. T_2_ maps were generated using custom written software (IDL; Exelis VIS, Herndon, VA) from three consecutive slices of the lower leg and thigh with the center slice being in the region in which the most proximal slice that the flexor digitorum longus (FDL) was visually present for the lower leg and the biceps femoris short head (BFs) for the thigh. The muscles of interest were carefully drawn within the borders to avoid any potential contamination of inter-muscular fascia [16].

^1^H-MRS measures of lipid fraction and T_2_ were performed for the Sol and VL using automated processing of spectra. Lipid fraction was assessed using area integration of the phase corrected spectra from the lipid (0.5–2.75 ppm) and ^1^H_2_O (4.3–5.10 ppm) region of the spectrum using custom written software (IDL; [20]). In order to minimize the impact of subject motion on data quality, spectra were stored dynamically (16 X 4 phase cycled averages) and outliers were omitted using an automated routine to remove spectra with a ^1^H_2_O peak height that deviated greater than 2.3 SD (99 percentile) from the mean. ^1^H_2_O and lipid signals were corrected for relaxation using the T_2_ of ^1^H_2_O measured for each subject, DMD group mean value of T_2_ of lipid acquired at 3T in a separate study [22], and literature values for T_1_ of lipid and ^1^H_2_O [28], [29].

The spectroscopic ^1^H_2_O T_2_ values were derived using the amplitude of the ^1^H_2_O signal at non-linear spaced echo times (TE’s; Sol: 11, 14, 18, 27, 36, 45, 54, 63, 81, 90, 108, 135, 162, 198, 243, and 288 ms; VL: 11,27,54, and 243 ms) using complex principal component analysis [30], [31]. T_2_ was determined by a non-linear curve fit to the decay in water signal as a function of TE using a mono-exponential model. For the acquisition from the Sol, in which 16 echoes were acquired, outliers (e.g., due to subject movement) were removed by excluding data points that were outside the 99% confidence interval of the fit.

### Statistical Methods

Comparisons between DMD and controls and among age groups were made using Wilcoxon rank sum test with a Bonferroni correction for the four age groups and for the six comparisons between age groups, respectively (Prism Software, GraphPad, v6.0b). The relationship between MRI-T_2_ and lipid/(lipid+water) was quantified using Spearman’s correlation coefficient. Statistical significance was defined as a p-value less than or equal to the Bonferroni corrected p-value of 0.05.

## Results

### Demographics

The descriptive characteristics of the boys with DMD and the control subjects are presented in Table 1. The control subjects were taller (p<0.01) in each age group and had lower body mass index (BMI; p<0.01) in the 11–14 year age group than DMD. The MR exam was well tolerated, and successful data were obtained from each exam. Out of the 154 subjects, one spectroscopy scan was not useable from the Sol and six were not useable from the VL. For the 2D SE scan, eight scans from the lower leg and 17 scans from the upper leg were not useable, mainly due to motion artifacts.

**Table 1:** Subject demographics of Duchenne muscular dystrophy (DMD) and unaffected control boys.

Age Group	Controls/DMD	Age (years)	Height (m)	Weight (kg)	BMI (kg/m^2^)
5–6.9 years	Controls (n = 6)	6.4 (0.6)	1.21 (0.10)	22.2 (6.1)	15.1 (2.0)
	DMD (n = 36)	6.1 (0.6)	1.10 (0.05)*	20.4 (3.0)	16.8 (1.7)
7–8.9 years	Controls (n = 5)	8.4 (0.3)	1.32 (0.05)	32.6 (9.7)	18.7 (5.1)
	DMD (n = 38)	7.9 (0.6)	1.21 (0.05)*	26.4 (5.2)	18.1 (3.1)
9–10.9 years	Controls (n = 12)	9.9 (0.6)	1.40 (0.08)	33.8 (8.5)	17.0 (2.6)
	DMD (n = 25)	9.9 (0.6)	1.26 (0.09)*	30.2 (6.9)	19.1 (3.3)
11–14 years	Controls (n = 8)	12.6 (1.6)	1.56 (0.06)	44.8 (11.0)	18.3 (3.3)
	DMD (n = 24)	11.9 (0.9)	1.32 (0.07)*	42.1 (9.8)	24.0 (4.8)*

*Denotes significantly different (p<0.05) than controls within age group. Values are mean (SD); Body mass index (BMI).

### Controls versus DMD within each age group

**Figure 2:**
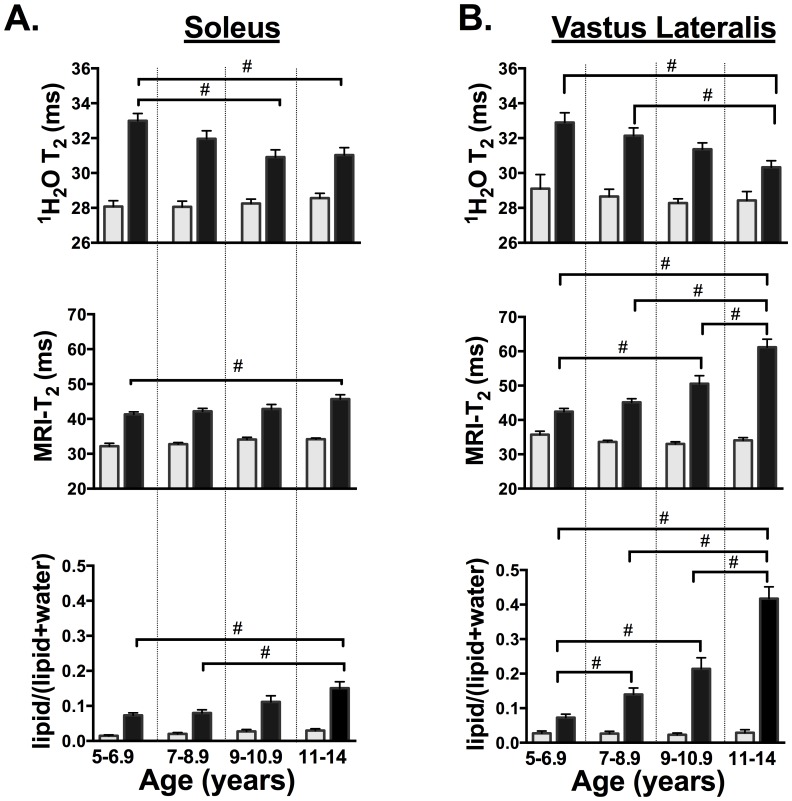
MRS ^1^H_2_O T_2_ (ms), MRI-T_2_ (ms), and lipid fraction [lipid/(lipid+water)] in the soleus (A) and vastus lateralis (B) of control and DMD age groups. DMD were significantly different (<0.05) than controls in all age groups, except lipid/(lipid+water) of the vastus lateralis in the 5–6.9 age group. # denotes significantly different (<0.05) between age groups in DMD. No differences were observed among age groups in controls. Bars represent mean (SEM).

Lower and upper leg axial SE images with single voxel ^1^H-MRS spectra were acquired from controls and boys with DMD at different ages showing disease progression (Fig. 1). MRI-T_2_ mapping and ^1^H_2_O T_2_ and lipid fraction derived using single voxel ^1^H-MRS were evaluated in the Sol and VL. In the Sol, MRI-T_2_, ^1^H_2_O T_2_, and lipid fraction were greater (p<0.05) in DMD compared to controls in each age group (5–6.9, 7–8.9, 9–10.9, 11–14 years; Fig. 2; Table 2). In the VL, MRI-T_2_, and ^1^H_2_O T_2_ were greater (p<0.05) in DMD compared to controls in every age group and lipid fraction was greater in DMD than controls in the 7–8.9, 9–10.9, and 11–14 year age groups (Fig. 2). Table 2 shows the values and statistical significance (p value) of the MR measures between controls and DMD subjects in the youngest age group (5–6.9 years).

**Table 2:** Comparison of MR measures between controls and DMD subjects at 5–6.9. years of age in the Soleus (Sol) and vastus lateralis (VL).

	Controls (5–6.9 years, n = 6)	DMD (5–6.9 years, n = 36)	P value
Mean MRS fat fraction [Sol]	0.015 (0.006)	0.073 (0.042)	<0.001
Mean MRI T_2_ (ms) [Sol]	32.2 (1.9)	41.3 (4.3)	<0.001
Mean MRS^1^H_2_O T_2_ (ms) [Sol]	28.1 (0.81)	33.0 (2.4)	<0.001
Mean MRS fat fraction [VL]	0.027 (0.017)	0.073 (0.059)	0.182
Mean MRI T_2_ (ms) [VL]	35.7 (2.3)	42.4 (4.9)	0.001
Mean MRS ^1^H_2_O T_2_ (ms) [VL]	29.1 (1.8)	32.9 (3.2)	0.023

Values are mean (SD).

**Figure 3:**
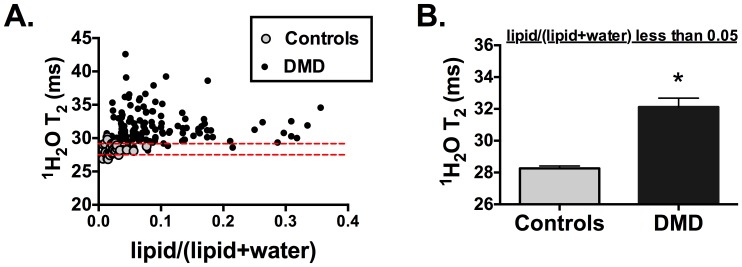
Scatterplot displaying the relationship between ^1^H_2_O T_2_ and lipid/(lipid+water) in the soleus of controls and boys with DMD. Red dotted lines denote 95% confidence interval of MRS ^1^H_2_O T_2_ in controls (A). In those with low lipid/(lipid+water) levels (i.e., less than 5%), the ^1^H_2_O T_2_ was longer in DMD (n = 34) than controls (n = 29) (B). * denotes significantly different (<0.05) than controls. Bars represent mean (SEM).

The relationship between ^1^H_2_O T_2_ and lipid/(lipid+water) in the Sol of controls and boys with DMD is displayed as a scatterplot (Fig. 3A). There was not a significant relationship between ^1^H_2_O T_2_ and lipid/(lipid+water) in DMD when subjects of all ages were included (r = −0.08, p = 0.361). Notably, muscle ^1^H_2_O T_2_ was greater in DMD than controls even in subjects with low muscle lipid fraction (i.e., in those with lipid/(lipid+water) less than 0.05; Fig. 3B). However, there was a strong relationship between MRI-T_2_ and lipid fraction in both the Sol (r = 0.74, p<0.0001) and VL (r = 0.92, p<0.0001) when subjects of all ages were included.

### Comparison of age groups

In control subjects, no differences (p>0.05) were observed in MRI-T_2_, ^1^H_2_O T_2_, and lipid fraction among the age groups (Fig. 2). In the boys with DMD, MRI-T_2_ and lipid fraction were greater (p<0.05) in the oldest age group (11–14 years) than in the youngest age group (5–6.9 years), whereas ^1^H_2_O T_2_ was lower in the oldest age group than in the youngest age group (Fig. 2). Also, the Sol and VL presented with several other differences among age groups in the MR measures (Fig. 2). Notably, the VL presented with elevations in lipid fraction from the 5–6.9 year age group to the 7–8.9 year old age group (Fig. 2).

### Comparison of lower extremity muscles

**Figure 4:**
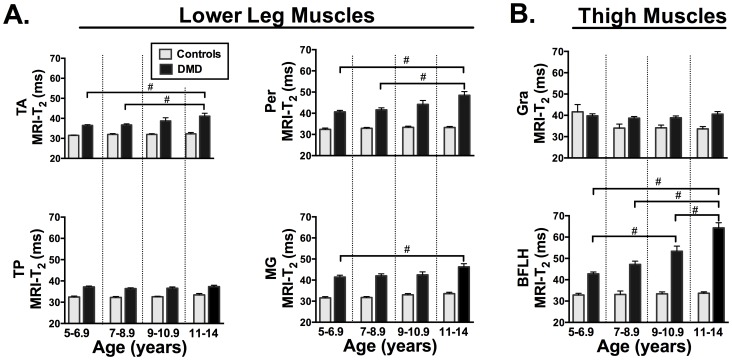
Comparison of tibialis anterior (TA), tibialis posterior (TP), peroneus brevis and longus (Per), and medial gastrocnemius (MG) of the lower leg (A) and the gracilis (Gra) and biceps femoris long head (BFLH) of the thigh (B). In all muscles and age groups DMD was greater than controls, except in Gra in the 5–6.9 and 7–8.9 age groups. # indicates differences (<0.05) among age groups in DMD. No significant differences were observed among control age groups. Bars represent mean (SEM).

Using MRI-T_2_ mapping, considerable differences among muscles were observed in DMD (Figs. 2 and 4). For example, the TP and Gra presented with no changes (p>0.05) among the age groups examined in this study, whereas the BFLH and VL progressed rapidly with increasing age in DMD. Also, the TA, Per, MG, and Sol were observed to be different between the young (5–6.9 years) and oldest age group (11–14 years) in DMD.

## Discussion

This study evaluated lower extremity skeletal muscles in a large cohort of ambulatory boys with DMD and unaffected controls in a range of age groups using MR measures of T_2_ and lipid fraction in a cross sectional design. The main findings of this study were that: 1) ^1^H_2_O T_2_, MRS determined muscle lipid fraction, and MRI-T_2_ were elevated (p<0.05) in skeletal muscles of DMD compared to controls in every age group examined, including in the youngest age group (5–6.9 years); 2) ^1^H_2_O T_2_ decreased with age groups in DMD; 3) MRI-T_2_ and muscle lipid fraction increased with age groups in several lower extremity muscles; and 4) the MRI-T_2_ values were strongly associated with muscle lipid fraction measured with ^1^H-MRS. Overall, these results demonstrate that muscle ^1^H_2_O T_2_, MRS determined lipid fraction, and MRI-T_2_ are sensitive to muscle involvement in DMD, including in young boys (5–6.9 years).

### Muscle damage and Inflammation/edema in Dystrophic Muscle

Skeletal muscles of DMD are characterized by impaired sarcolemma integrity, increased susceptibility to muscle damage [23], [32], and inflammation/edema [33], [34]. Factors associated with muscle damage and inflammation have been directly related to an increase in ^1^H_2_O T_2_ [23], [35], [36]. In this study, we observed an elevated ^1^H_2_O T_2_, derived using single voxel ^1^H-MRS, a method that provides high fidelity spectral separation of water and lipid MR signals, in the Sol and VL of boys with DMD compared to controls.

The magnitude of the difference in ^1^H_2_O T_2_ between DMD and controls was, on average, greatest in the youngest boys, while ^1^H_2_O T_2_ decreased with age in DMD. For example, the 9–10.9 year age group was decreased compared to the 5–6.9 years in the soleus and the 11–14 year age group was decreased compared to the 5–6.9 year age group in the VL. The decrease in ^1^H_2_O T_2_ at the older ages relative to the younger ages may be due to less inflammation (in part, or whole, due to the increased incidence of steroid use in older boys [37]) and increased fibrosis [38], [39], [40]. Importantly, this study also demonstrates that ^1^H_2_O T_2_ is increased in those DMD subjects that have minimal lipid infiltration in skeletal muscle (less than 5%), suggesting T_2_ may be a valuable marker of muscle damage and inflammation/edema in young subjects of 5–6.9 years, prior to fatty tissue infiltration.

The elevated ^1^H_2_O T_2_ in DMD compared to controls is in agreement with previous findings from our laboratory in which MRI-T_2_ with fat saturation (resulting in minimal contribution of lipid) was elevated in 5–8 year old boys with DMD compared to controls [16]. In support of this, elevated signal intensity in T_2_-STIR images was associated with increased immune and inflammatory cells (CD8+ T cells, IL12p40, IFNγ and TNFα) in facioscapulohumeral muscular dystrophy [35]. However, while fat saturation reduces the lipid contribution to T_2_, it does not completely eliminate the contribution of lipid with T_2_ mapping. Therefore, spectroscopic relaxometry, as used in this study, has an advantage of resolving the major individual lipid resonances from water based on chemical shifts.

### Fatty tissue Infiltration in Dystrophic Muscle

In contrast to ^1^H_2_O T_2_ decreasing with age in DMD, MRI-T_2_ from T_2_ mapping either increased or did not change with age, depending on the muscle group. The increase in MRI-T_2_ in the Sol and VL was tightly coupled with the increase in muscle lipid fraction, as shown in other studies [14], [41]. This indicates that MRI signal associated with fatty tissue infiltration is the primary pathological constituent responsible for the increase in MRI-T_2_ observed with older ages in DMD. Progressive lipid infiltration as assessed by MRI 3-point Dixon [17], [18] and single voxel ^1^H-MRS [19] has been associated with disease progression, age, and deterioration of clinical functional performance in DMD. However, it should be appreciated that since ^1^H_2_O T_2_ decreases a relatively small, but measurable, amount with age, this may partially negate the increases in MRI-T_2_ that occur due to lipid infiltration. For example, in this study, it is possible that MRI-T_2_ could have been utilized to detect earlier changes if lipid and water T_2_ contributions to overall MRI-T_2_ were separated. New developments in MR acquisition and analysis have recently been proposed that enable an estimate of the water and fat contribution to MRI T_2_ [14], [42], and these methods may be valuable in future studies. As a result, while T_2_ mapping has the potential to be influenced by both ^1^H_2_O T_2_ and lipid infiltration, the relative contribution of each to the elevated MRI-T_2_ in DMD likely varies considerably with age and disease progression. For example, ^1^H_2_O T_2_ may be expected to be relatively more influential on MRI-T_2_ at a younger age before significant lipid infiltration.

### Potential of MR as outcome measures

The results of this study support muscle MRI-T_2_, ^1^H_2_O T_2_, and lipid fraction being used to monitor disease involvement in DMD, including in boys at a young age. These MR measures have recently been shown to have an excellent day-to-day reproducibility in subjects with DMD as well as across sites [21], and have the advantage of being less dependent on motivation, attention, and coordination than functional tests [9]. Furthermore, either the MRS or MRI measures enable multiple muscles to be evaluated within a relatively short amount of time. In this study, the VL presented with earlier increases in the youngest age groups compared to the Sol, indicating the VL muscle accumulates pathology faster than the Sol. T_2_ mapping and other imaging sequences, such as 3-Point Dixon [22], have an advantage of greater coverage and information about more muscle groups in a similar amount of acquisition time as single voxel ^1^H-MRS (Table S1). With T_2_ mapping there was further heterogeneity observed of different muscle groups, with the Gra, TA, and TP showing less change and the BFLH increasing at the fastest rate of the muscles analyzed in this study. Future studies would benefit from comparing the relationship of these MR measures with functional tests in a large cohort, as previously done in smaller cohorts and other dystrophies [16], [20], [43].

Therefore, the optimal muscle groups to target and the MR measures to acquire will likely depend on the stage of disease progression and the potential therapeutic intervention. For example, ^1^H_2_O T_2_ may be a valuable measure for examining the effects of anti-inflammatory treatments in DMD. Other advantages of using MR to monitor disease involvement in DMD are that the measures are not limited to ambulatory patients and they have the potential to be performed in children younger than five years, particularly when motion correction strategies are implemented.

## Summary

In this study ^1^H_2_O T_2_ derived using ^1^H-MRS and MRI-T_2_ were observed to be sensitive to DMD associated pathologies, consistent with increased muscle damage and inflammation/edema. Furthermore, muscle MRI-T_2_ increased with disease progression in DMD and was strongly associated with progressive lipid infiltration as assessed by ^1^H-MRS. Overall, this study supports that MR measures of muscle T_2_ and lipid fraction may be sensitive to disease involvement and potential therapeutic interventions in DMD in all age groups, including younger boys.

## Supporting Information

Table S1 Comparison of coverage in foot head direction and scan time among MR acquisitions used in this study and a 3-point Dixon scan used previously.
(DOCX)
