# Correction: Magnetic Resonance Imaging and Spectroscopy Assessment of Lower Extremity Skeletal Muscles in Boys with Duchenne Muscular Dystrophy: A Multicenter Cross Sectional Study

**DOI:** 10.1371/journal.pone.0111822

**Published:** 2014-10-22

**Authors:** The PLOS ONE Staff

## Notice of Republication

This article was republished on September 24, 2014, due to Table S1 being erroneously removed. The publisher apologizes for this error. Please view this article again for the Supporting Information file. The originally published, uncorrected article and the republished, corrected article are provided here for reference.

## Supporting Information

File S1 **Originally published, uncorrected article**
(PDF)

File S2 **Republished, corrected article**
(PDF)
