# Supplementary material for: Magnetic Resonance Imaging and Spectroscopy Assessment of Lower Extremity Skeletal Muscles in Boys with Duchenne Muscular Dystrophy: A Multicenter Cross Sectional Study
Source: PLoS One. 2014 Sep 9;9(9):e106435. doi: 10.1371/journal.pone.0106435 (PMC4159278; doi:10.1371/journal.pone.0106435)
Supplement: Table S1 — Comparison of coverage in foot head direction and scan time among MR acquisitions used in this study and a 3-point Dixon scan used previously. (DOCX) [file pone.0106435.s001.docx]

**Table S1.** Comparison of coverage in foot head direction and scan time among MR

acquisitions used in this study and a 3-point Dixon scan used previously.

|  | **Coverage in foot head direction** | **Scan Time (including shim and preparation time)** |
| --- | --- | --- |
| MRS lipid fraction | ~4 cm (10X20X40mm) | 3.5 minutes (64X9 s TR) |
| MRS ^1^H_2_O T_2_ | ~4 cm (10X20X40mm) | 5 minutes (4 echoes, 8 averages) or 10 minutes (16 echoes, 4 averages) |
| 2D Spin Echo | 4.2-8.4 cm (4-8 slices, 7mm slice thickness, 3.5 mm gap) | 4-6 min |
| Dixon Imaging [as acquired in ref. [22](#_ENREF_22)] | 8-12.5 cm (16-25 slices, 4 mm slice thickness, 1mm gap) | 3-5 min |
