# Supplementary material for: Correction: Magnetic Resonance Imaging and Spectroscopy Assessment of Lower Extremity Skeletal Muscles in Boys with Duchenne Muscular Dystrophy: A Multicenter Cross Sectional Study
Source: PLoS One. 2014 Oct 22;9(10):e111822. doi: 10.1371/journal.pone.0111822 (PMC4206487; doi:10.1371/journal.pone.0111822)
Supplement: File S2 — Republished, corrected article (PDF) [file pone.0111822.s002.pdf]

Magnetic Resonance Imaging and Spectroscopy Assessment of Lower Extremity Skeletal Muscles in Boys with Duchenne Muscular Dystrophy: A Multicenter Cross Sectional Study

Sean C. Forbes[1]*, Rebecca J. Willcocks[1], William T. Triplett[1], William D. Rooney[2], Donovan J. Lott[1], Dah-Jyuu Wang[3], Jim Pollaro[2], Claudia R. Senesac[1], Michael J. Daniels[4], Richard S. Finkel[3,5], Barry S. Russman[2], Barry J. Byrne[6], Erika L. Finanger[2], Gihan I. Tennekoon[3], Glenn A. Walter[7], H. Lee Sweeney[8], Krista Vandenborne[1]

1 Department of Physical Therapy, University of Florida, Gainesville, FL, United States of America, **2** Oregon Health & Science University, Portland, OR, United States of America, **3** The Children's Hospital of Philadelphia, Philadelphia, PA, United States of America, **4** Department of Statistics & Data Sciences and Department of Integrative Biology, the University of Texas at Austin, Austin, TX, United States of America, **5** Nemours Children's Hospital, Orlando, Florida, United States of America, **6** Department of Pediatrics and Molecular Genetics & Microbiology, Powell Gene Therapy Center, University of Florida, Gainesville, FL, United States of America, **7** Department of Physiology and Functional Genomics, University of Florida, Gainesville, FL, United States of America, **8** Department of Physiology, University of Pennsylvania, Philadelphia, PA, United States of America

Abstract

Introduction: Duchenne muscular dystrophy (DMD) is an X-linked recessive disorder that results in functional deficits. However, these functional declines are often not able to be quantified in clinical trials for DMD until after age 7. In this study, we hypothesized that ^1H_2O T_2 derived using ^{1}H-MRS and MRI-T_2 will be sensitive to muscle involvement at a young age (5–7 years) consistent with increased inflammation and muscle damage in a large cohort of DMD subjects compared to controls.

Methods: MR data were acquired from 123 boys with DMD (ages 5–14 years; mean 8.6 SD 2.2 years) and 31 healthy controls (age 9.7 SD 2.3 years) using 3-Tesla MRI instruments at three institutions (University of Florida, Oregon Health & Science University, and Children's Hospital of Philadelphia). T_2-weighted multi-slice spin echo (SE) axial images and single voxel ^{1}H-MRS were acquired from the lower leg and thigh to measure lipid fraction and ^1H_2O T_2.

Results: MRI-T_2, ^1H_2O T_2, and lipid fraction were greater ($p<0.05$) in DMD compared to controls. In the youngest age group, DMD values were different ($p<0.05$) than controls for the soleus MRI-T_2, ^1H_2O T_2 and lipid fraction and vastus lateralis MRI-T_2 and ^1H_2O T_2. In the boys with DMD, MRI-T_2 and lipid fraction were greater ($p<0.05$) in the oldest age group (11–14 years) than the youngest age group (5–6.9 years), while ^1H_2O T_2 was lower in the oldest age group compared to the young age group.

Discussion: Overall, MR measures of T_2 and lipid fraction revealed differences between DMD and Controls. Furthermore, MRI-T_2 was greater in the older age group compared to the young age group, which was associated with higher lipid fractions. Overall, MR measures of T_2 and lipid fraction show excellent sensitivity to DMD disease pathologies and potential therapeutic interventions in DMD, even in the younger boys.

Citation: Forbes SC, Willcocks RJ, Triplett WT, Rooney WD, Lott DJ, et al. (2014) Magnetic Resonance Imaging and Spectroscopy Assessment of Lower Extremity Skeletal Muscles in Boys with Duchenne Muscular Dystrophy: A Multicenter Cross Sectional Study. PLoS ONE 9(9): e106435. doi:10.1371/journal.pone.0106435

Editor: Reshma Taneja, National University of Singapore, Singapore

Received May 19, 2014; **Accepted** July 29, 2014; **Published** September 9, 2014

Copyright: © 2014 Forbes et al. This is an open-access article distributed under the terms of the Creative Commons Attribution License, which permits unrestricted use, distribution, and reproduction in any medium, provided the original author and source are credited.

Data Availability: The authors confirm that all data underlying the findings are fully available without restriction. All relevant data are within the paper.

Funding: This study was supported by National Institute of Arthritis and Musculoskeletal and Skin Diseases/National Institute of Neurological Disorders and Stroke R01AR056973/R01AR065943 and Wellstone Muscular Dystrophy Center 1U54AR052646. The funders had no role in study design, data collection and analysis, decision to publish, or preparation of the manuscript.

Competing Interests: R.S.F. Financial activities related to the present article: None to disclose. Financial activities not related to the present article: institution received a grant from PTC Therapeutics for a study of ataluren in DMD for which R.S.F. was the primary investigator (some of the subjects in that study also participated in the current study, but no financial conflict is identified). Other relationships: unpaid advisor to Muscular Dystrophy Association and Parent Project Muscular Dystrophy. This does not alter our adherence to PLOS ONE policies on sharing data and materials.

* Email: scforbes@ufl.edu

Introduction

Duchenne muscular dystrophy (DMD) is an X-linked recessive disorder caused by mutations in the dystrophin gene [1]. DMD has an incidence of 1 in 3,600–6,000 male births and is characterized by progressive muscle deterioration, loss of functional abilities, and reduced life expectancy [2]. Symptoms of

DMD are usually recognized between two and three years of age, and include delayed walking and running, and difficulty climbing stairs [2]. Boys with DMD are typically diagnosed by five years of age [3,4].

Currently there is no cure for DMD, although there are a number of therapeutic interventions that have shown promise in preclinical and early clinical trials, including viral delivery of microdystrophin genes [5], exon skipping [6], and small molecule therapies, such as ataluren [7]. Interventions for boys with DMD may be most effective in young boys with DMD who have not yet experienced significant muscle deterioration and atrophy [8]. Therefore, there is a need for sensitive biomarkers to measure muscle involvement to evaluate the effects of potential therapeutic interventions in young boys with DMD.

The six minute walk test (6MWT) has been utilized as the primary outcome measure in two recent phase IIb/III clinical trials in DMD [9]. However, functional decline in motor performance, such as reduced distance walked in the 6MWT, is often not observed in DMD until after age seven [10]. Furthermore, the 6MWT has been criticized for its dependence on subject attention span, motivation, and neuromuscular coordination [9]. In addition to the 6MWT, comprehensive motor evaluations have been proposed using a battery of functional tests, such as the North Star Ambulatory Assessment, which uses a composite score of 17 items [11,12]. This score was recently shown to be sensitive to detect differences between corticosteroid regimens in DMD [13].

Magnetic resonance imaging (MRI) and spectroscopy (MRS) may also have the potential to be sensitive markers of muscle involvement in DMD. The MRI transverse relaxation time constant (MRI-T_2) that represents the overall bulk T_2 of the region of interest, and is influenced by both lipid and ^1H_2O components [14,15], has previously been observed to differentiate boys with DMD from controls [16]. Furthermore, lipid infiltration has been associated with disease progression, age, and clinical functional tests in DMD as assessed using MRI 3-point Dixon [17,18] and single voxel ^{1}H-MRS [19,20]. Also, ^1H_2O T_2 of skeletal muscles assessed using single voxel ^{1}H-MRS, a measure independent of lipid infiltration, has been shown to be greater in DMD compared to controls [21,22], indicating elevated muscle damage and inflammation/edema [23]. As a result, lipid fraction, ^1H_2O T_2, and MRI-T_2 all have the potential to be effective in monitoring disease progression in DMD. In a previous study, we have shown that using standardized procedures these quantitative MR measures of muscle composition can be reproducibly implemented within and across multiple sites [21].

Therefore, using a multicenter trial design with a large cohort of ambulatory boys with DMD and controls of different age groups (5–6.9, 7–8.9, 9–10.9, and 11–14 years) we measured lipid fraction, ^1H_2O T_2, and MRI-T_2 of muscles in the lower and upper leg to assess the potential of these measures to monitor muscle involvement in DMD and ultimately test potential therapeutic interventions in future studies. We hypothesized that: 1) ^1H_2O T_2 is sensitive to muscle disease, even at a young age (5–6.9 years), consistent with increased muscle damage and inflammation/edema in DMD compared to controls; 2) ^1H_2O T_2 and MRI-T_2 will be altered with age and disease progression; and 3) MRI-T_2 will be associated with lipid fraction and both MRI-T_2 and lipid fraction will increase with age groups in lower and upper leg muscles of DMD.

Methods

The data from this study were collected as a part of the multi-center Imaging DMD study (http://imagingdmd.org/) ([21,22,24]). The study was approved by the Institutional Review Boards (IRB) at the University of Florida (UF), Oregon Health & Science University (OHSU), and Children's Hospital of Philadelphia (CHOP). The study was in compliance with the Health Insurance Portability and Accountability Act (HIPAA) and an informed written assent/consent was obtained from the subject/guardian prior to participation in the study.

Participants

MR data were acquired from 123 boys with DMD (ages 5–14 years; mean 8.6 SD 2.2 years) and 31 healthy controls (ages 5–14 years; age 9.7 SD 2.3). The boys with DMD were confirmed by genetic testing, ambulatory, and 86 were receiving corticosteroids. The controls were similar in age to the boys with DMD. All participants were asked to avoid any excessive physical activity beyond their normal levels for three days prior to the study.

MR acquisition

MR data were acquired from the lower leg and thigh from three centers by using a 3T Achieva Quasar Dual MRI instrument (Philips, Best, the Netherlands), a 3T Magnetom TIM Trio MRI instrument (Siemens, Erlangen, Germany), or a 3T Magnetom Verio MRI instrument (Siemens), as previously described [21]. Radiofrequency coil configurations differed between centers. For the lower leg, a transmit-receive quadrature extremity coil or an eight-channel sensitivity encoding volume receive-only knee coil was used. For the thigh, a two-channel surface coil, a body matrix array coil, or a transmit-receive quadrature extremity coil was used.

Subjects lay supine in the bore of the magnet with the leg secured using foam padding and rice bags. MRI/MRS measurements were collected from the upper and lower regions of the right leg. T_2-weighted multi-slice spin echo (SE) axial images were acquired (4–6 slices, 7 mm slices, 3.5 mm gap; 16 TE's, 20–320 ms evenly spaced; TR = 3 s) (Fig. 1). The refocusing pulse width was set at 1.5 times the excitation pulse width on all scanners. Single voxel ^{1}H-MRS data were acquired (TE = 108 ms; TR = 3 s; NA = 64) for assessment of lipid fraction using stimulated-echo acquisition mode (STEAM) from the soleus (Sol) and vastus lateralis (VL; Fig. 1) [25,26]. Finally, ^{1}H spectroscopic relaxometry was performed using STEAM in the Sol (16 TE's non-linearly spaced from 11–288 ms; TR = 9 s; NA = 4) and VL (4 TE's non-linearly spaced from 11–243 ms; TR = 9 s; NA = 4). The total acquisition time including subject set-up was approximately 75 minutes.

MR Analysis

The MR data were transferred to and analyzed at a single center. Imaging-based T_2 values were measured for the tibialis anterior (TA), tibialis posterior (TP), peroneus longus and brevis (Per), soleus (Sol), medial gastrocnemius (MG), biceps femoris long head (BFLH), vastus lateralis (VL), and gracilis (Gra). Each of these measures used an average of 3 contiguous axial slices. T_2 maps were calculated for lower leg and thigh muscles by voxel-wise estimation of T_2 by fitting a single exponential equation to the magnitude signal intensity (S) from spin-echo images with TE values from 40–100 ms; $S(TE) = S_0 * \exp(-TE/T_2)$ by varying S_0 and T_2 [16,21]. The initial TE was not included to minimize the effect of stimulated echoes on the calculation of T_2 [27]. T_2 maps were generated using custom written software (IDL; Exelis VIS,

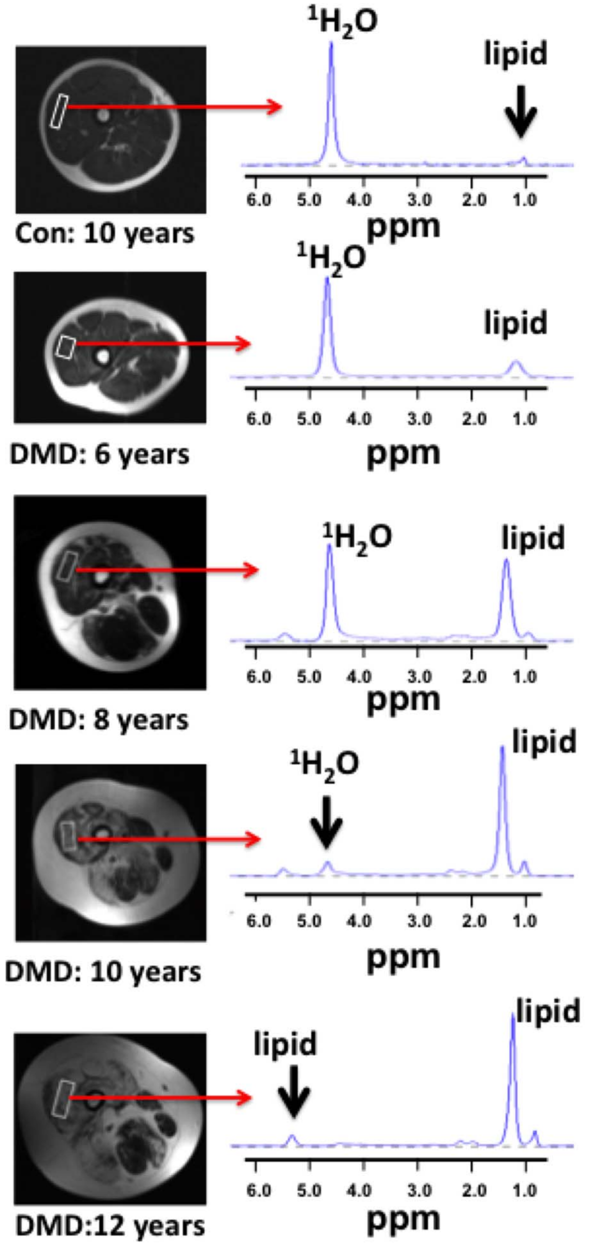

Figure 1. Example upper leg axial spin echo (SE) images (TE 60 ms) with single voxel ^{1}H-MRS spectra (TE 108 ms) from the vastus lateralis of a control and boys with DMD at different ages.
doi:10.1371/journal.pone.0106435.g001

Herndon, VA) from three consecutive slices of the lower leg and thigh with the center slice being in the region in which the most proximal slice that the flexor digitorum longus (FDL) was visually present for the lower leg and the biceps femoris short head (BFs) for the thigh. The muscles of interest were carefully drawn within the borders to avoid any potential contamination of inter-muscular fascia [16].

^{1}H-MRS measures of lipid fraction and T_2 were performed for the Sol and VL using automated processing of spectra. Lipid fraction was assessed using area integration of the phase corrected spectra from the lipid (0.5–2.75 ppm) and ^1H_2O (4.3–5.10 ppm) region of the spectrum using custom written software (IDL; [20]). In order to minimize the impact of subject motion on data quality, spectra were stored dynamically (16 X 4 phase cycled averages) and outliers were omitted using an automated routine to remove spectra with a ^1H_2O peak height that deviated greater than 2.3 SD (99 percentile) from the mean. ^1H_2O and lipid signals were corrected for relaxation using the T_2 of ^1H_2O measured for each subject, DMD group mean value of T_2 of lipid acquired at 3T in a separate study [22], and literature values for T_1 of lipid and ^1H_2O [28,29].

The spectroscopic ^1H_2O T_2 values were derived using the amplitude of the ^1H_2O signal at non-linear spaced echo times (TE's; Sol: 11, 14, 18, 27, 36, 45, 54, 63, 81, 90, 108, 135, 162, 198, 243, and 288 ms; VL: 11,27,54, and 243 ms) using complex principal component analysis [30,31]. T_2 was determined by a non-linear curve fit to the decay in water signal as a function of TE using a mono-exponential model. For the acquisition from the Sol, in which 16 echoes were acquired, outliers (e.g., due to subject movement) were removed by excluding data points that were outside the 99% confidence interval of the fit.

Statistical Methods

Comparisons between DMD and controls and among age groups were made using Wilcoxon rank sum test with a Bonferroni correction for the four age groups and for the six comparisons between age groups, respectively (Prism Software, GraphPad, v6.0b). The relationship between MRI-T_2 and lipid/(lipid+water) was quantified using Spearman's correlation coefficient. Statistical significance was defined as a p-value less than or equal to the Bonferroni corrected p-value of 0.05.

Results

Demographics

The descriptive characteristics of the boys with DMD and the control subjects are presented in Table 1. The control subjects were taller ($p<0.01$) in each age group and had lower body mass index (BMI; $p<0.01$) in the 11–14 year age group than DMD. The MR exam was well tolerated, and successful data were obtained from each exam. Out of the 154 subjects, one spectroscopy scan was not useable from the Sol and six were not useable from the VL. For the 2D SE scan, eight scans from the lower leg and 17 scans from the upper leg were not useable, mainly due to motion artifacts.

Controls versus DMD within each age group

Lower and upper leg axial SE images with single voxel ^{1}H-MRS spectra were acquired from controls and boys with DMD at different ages showing disease progression (Fig. 1). MRI-T_2 mapping and ^1H_2O T_2 and lipid fraction derived using single voxel ^{1}H-MRS were evaluated in the Sol and VL. In the Sol, MRI-T_2, ^1H_2O T_2, and lipid fraction were greater ($p<0.05$) in DMD compared to controls in each age group (5–6.9, 7–8.9, 9–10.9, 11–14 years; Fig. 2; Table 2). In the VL, MRI-T_2, and ^1H_2O T_2 were greater ($p<0.05$) in DMD compared to controls in every age group and lipid fraction was greater in DMD than controls in the 7–8.9, 9–10.9, and 11–14 year age groups (Fig. 2). Table 2 shows the values and statistical significance (p value) of the MR measures between controls and DMD subjects in the youngest age group (5–6.9 years).

The relationship between ^1H_2O T_2 and lipid/(lipid+water) in the Sol of controls and boys with DMD is displayed as a scatterplot (Fig. 3A). There was not a significant relationship between ^1H_2O T_2 and lipid/(lipid+water) in DMD when subjects of all ages were included ($r=-0.08$, $p=0.361$). Notably, muscle ^1H_2O T_2 was

Table 1. Subject demographics of Duchenne muscular dystrophy (DMD) and unaffected control boys.

Age Group	Controls/DMD	Age (years)	Height (m)	Weight (kg)	BMI (kg/m^2)
5–6.9 years	Controls (n = 6)	6.4 (0.6)	1.21 (0.10)	22.2 (6.1)	15.1 (2.0)
	DMD (n = 36)	6.1 (0.6)	1.10 (0.05)*	20.4 (3.0)	16.8 (1.7)
7–8.9 years	Controls (n = 5)	8.4 (0.3)	1.32 (0.05)	32.6 (9.7)	18.7 (5.1)
	DMD (n = 38)	7.9 (0.6)	1.21 (0.05)*	26.4 (5.2)	18.1 (3.1)
9–10.9 years	Controls (n = 12)	9.9 (0.6)	1.40 (0.08)	33.8 (8.5)	17.0 (2.6)
	DMD (n = 25)	9.9 (0.6)	1.26 (0.09)*	30.2 (6.9)	19.1 (3.3)
11–14 years	Controls (n = 8)	12.6 (1.6)	1.56 (0.06)	44.8 (11.0)	18.3 (3.3)
	DMD (n = 24)	11.9 (0.9)	1.32 (0.07)*	42.1 (9.8)	24.0 (4.8)*

*Denotes significantly different ($p<0.05$) than controls within age group. Values are mean (SD); Body mass index (BMI).
doi:10.1371/journal.pone.0106435.t001

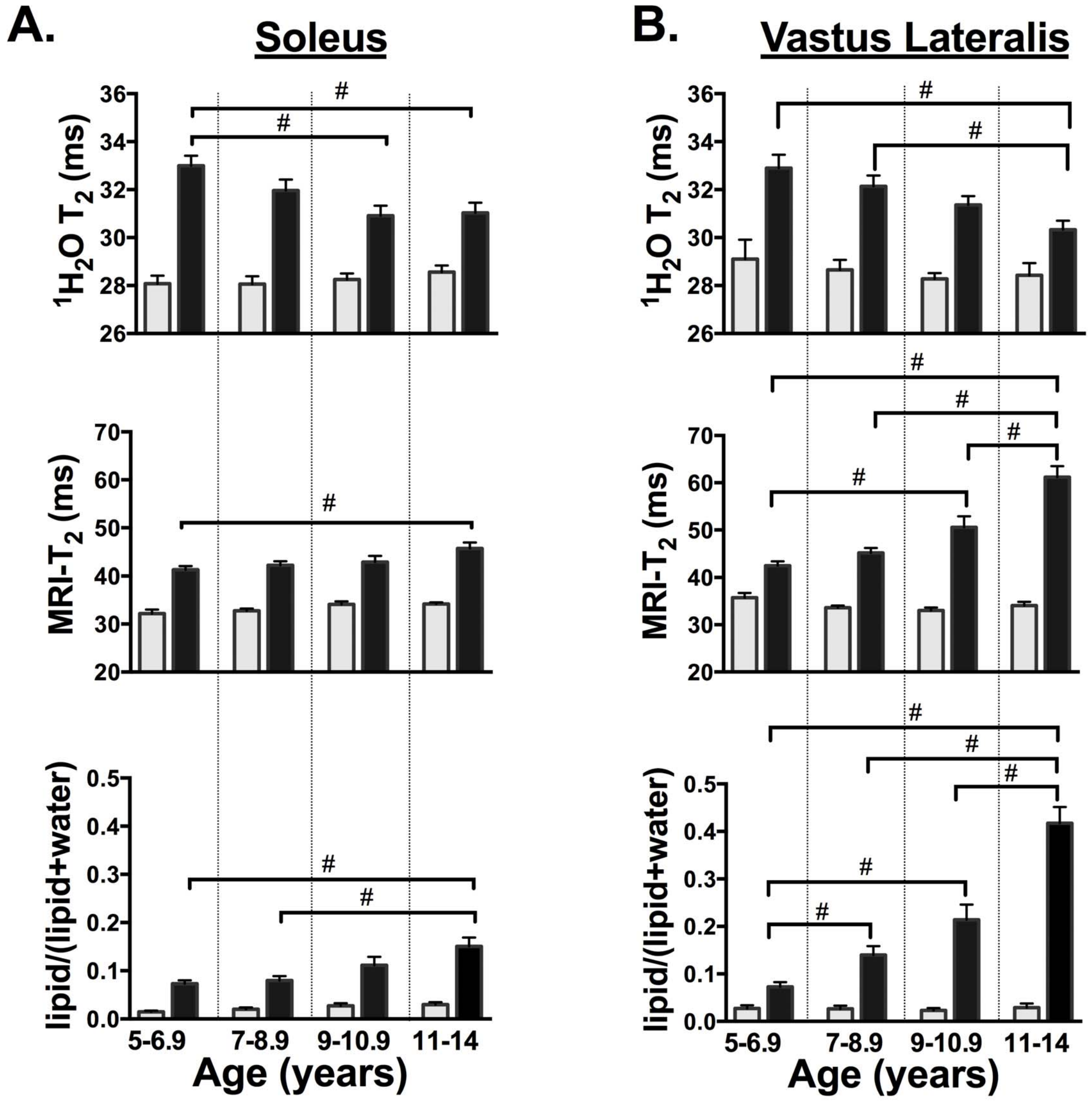

Figure 2. MRS ^1H_2O T_2 (ms), MRI-T_2 (ms), and lipid fraction [lipid/(lipid+water)] in the soleus (A) and vastus lateralis (B) of control and DMD age groups. DMD were significantly different (<0.05) than controls in all age groups, except lipid/(lipid+water) of the vastus lateralis in the 5–6.9 age group. # denotes significantly different (<0.05) between age groups in DMD. No differences were observed among age groups in controls. Bars represent mean (SEM).
doi:10.1371/journal.pone.0106435.g002

Table 2. Comparison of MR measures between controls and DMD subjects at 5–6.9. years of age in the Soleus (Sol) and vastus lateralis (VL).

	Controls (5–6.9 years, n = 6)	DMD (5–6.9 years, n = 36)	P value
Mean MRS fat fraction [Sol]	0.015 (0.006)	0.073 (0.042)	<0.001
Mean MRI T_2 (ms) [Sol]	32.2 (1.9)	41.3 (4.3)	<0.001
Mean MRS ^1H_2O T_2 (ms) [Sol]	28.1 (0.81)	33.0 (2.4)	<0.001
Mean MRS fat fraction [VL]	0.027 (0.017)	0.073 (0.059)	0.182
Mean MRI T_2 (ms) [VL]	35.7 (2.3)	42.4 (4.9)	0.001
Mean MRS ^1H_2O T_2 (ms) [VL]	29.1 (1.8)	32.9 (3.2)	0.023

Values are mean (SD).
doi:10.1371/journal.pone.0106435.t002

greater in DMD than controls even in subjects with low muscle lipid fraction (i.e., in those with lipid/(lipid+water) less than 0.05; Fig. 3B). However, there was a strong relationship between MRI-T_2 and lipid fraction in both the Sol ($r = 0.74$, $p < 0.0001$) and VL ($r = 0.92$, $p < 0.0001$) when subjects of all ages were included.

Comparison of age groups

In control subjects, no differences ($p > 0.05$) were observed in MRI-T_2, ^1H_2O T_2, and lipid fraction among the age groups (Fig. 2). In the boys with DMD, MRI-T_2 and lipid fraction were greater ($p < 0.05$) in the oldest age group (11–14 years) than in the youngest age group (5–6.9 years), whereas ^1H_2O T_2 was lower in the oldest age group than in the youngest age group (Fig. 2). Also, the Sol and VL presented with several other differences among age groups in the MR measures (Fig. 2). Notably, the VL presented with elevations in lipid fraction from the 5–6.9 year age group to the 7–8.9 year old age group (Fig. 2).

Comparison of lower extremity muscles

Using MRI-T_2 mapping, considerable differences among muscles were observed in DMD (Figs. 2 and 4). For example, the TP and Gra presented with no changes ($p > 0.05$) among the age groups examined in this study, whereas the BFLH and VL progressed rapidly with increasing age in DMD. Also, the TA, Per, MG, and Sol were observed to be different between the young (5–6.9 years) and oldest age group (11–14 years) in DMD.

Discussion

This study evaluated lower extremity skeletal muscles in a large cohort of ambulatory boys with DMD and unaffected controls in a range of age groups using MR measures of T_2 and lipid fraction in a cross sectional design. The main findings of this study were that: 1) ^1H_2O T_2, MRS determined muscle lipid fraction, and MRI-T_2 were elevated ($p < 0.05$) in skeletal muscles of DMD compared to controls in every age group examined, including in the youngest age group (5–6.9 years); 2) ^1H_2O T_2 decreased with age groups in DMD; 3) MRI-T_2 and muscle lipid fraction increased with age groups in several lower extremity muscles; and 4) the MRI-T_2 values were strongly associated with muscle lipid fraction measured with ^1H-MRS. Overall, these results demonstrate that muscle ^1H_2O T_2, MRS determined lipid fraction, and MRI-T_2 are sensitive to muscle involvement in DMD, including in young boys (5–6.9 years).

Muscle damage and Inflammation/edema in Dystrophic Muscle

Skeletal muscles of DMD are characterized by impaired sarcolemma integrity, increased susceptibility to muscle damage [23,32], and inflammation/edema [33,34]. Factors associated with muscle damage and inflammation have been directly related to an increase in ^1H_2O T_2 [23,35,36]. In this study, we observed an elevated ^1H_2O T_2, derived using single voxel ^1H-MRS, a method that provides high fidelity spectral separation of water and lipid

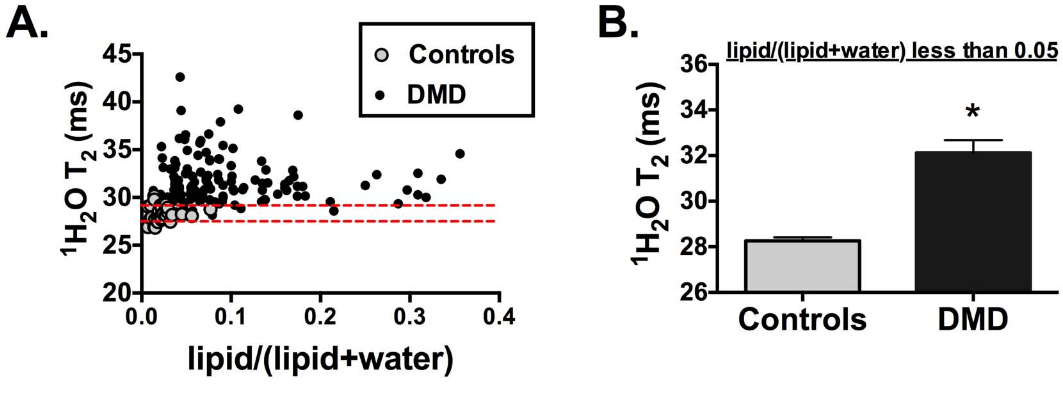

Figure 3. Scatterplot displaying the relationship between ^1H_2O T_2 and lipid/(lipid+water) in the soleus of controls and boys with DMD. Red dotted lines denote 95% confidence interval of MRS ^1H_2O T_2 in controls (A). In those with low lipid/(lipid+water) levels (i.e., less than 5%), the ^1H_2O T_2 was longer in DMD (n = 34) than controls (n = 29) (B). * denotes significantly different (<0.05) than controls. Bars represent mean (SEM).
doi:10.1371/journal.pone.0106435.g003

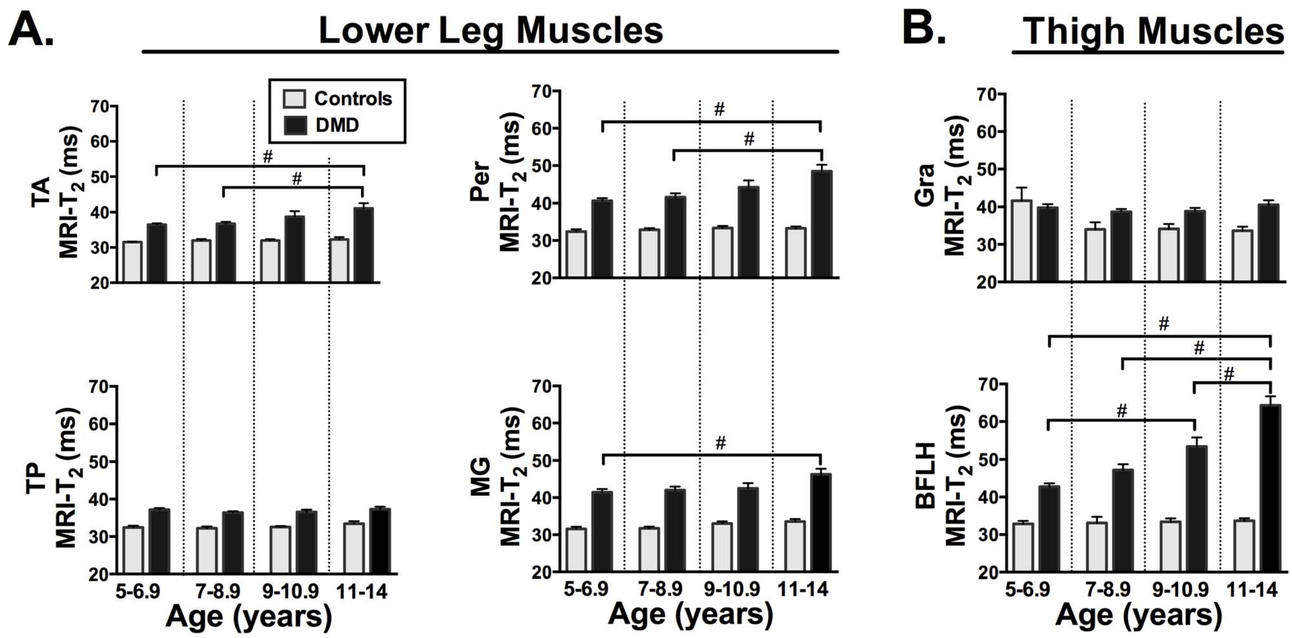

Figure 4. Comparison of tibialis anterior (TA), tibialis posterior (TP), peroneus brevis and longus (Per), and medial gastrocnemius (MG) of the lower leg (A) and the gracilis (Gra) and biceps femoris long head (BFLH) of the thigh (B). In all muscles and age groups DMD was greater than controls, except in Gra in the 5–6.9 and 7–8.9 age groups. # indicates differences (<0.05) among age groups in DMD. No significant differences were observed among control age groups. Bars represent mean (SEM).

doi:10.1371/journal.pone.0106435.g004

MR signals, in the Sol and VL of boys with DMD compared to controls.

The magnitude of the difference in ^1H_2O T_2 between DMD and controls was, on average, greatest in the youngest boys, while ^1H_2O T_2 decreased with age in DMD. For example, the 9–10.9 year age group was decreased compared to the 5–6.9 years in the soleus and the 11–14 year age group was decreased compared to the 5–6.9 year age group in the VL. The decrease in ^1H_2O T_2 at the older ages relative to the younger ages may be due to less inflammation (in part, or whole, due to the increased incidence of steroid use in older boys [37]) and increased fibrosis [38,39,40]. Importantly, this study also demonstrates that ^1H_2O T_2 is increased in those DMD subjects that have minimal lipid infiltration in skeletal muscle (less than 5%), suggesting T_2 may be a valuable marker of muscle damage and inflammation/edema in young subjects of 5–6.9 years, prior to fatty tissue infiltration.

The elevated ^1H_2O T_2 in DMD compared to controls is in agreement with previous findings from our laboratory in which MRI-T_2 with fat saturation (resulting in minimal contribution of lipid) was elevated in 5–8 year old boys with DMD compared to controls [16]. In support of this, elevated signal intensity in T_2-STIR images was associated with increased immune and inflammatory cells (CD8+ T cells, IL12p40, IFNγ and TNFα) in facioscapulohumeral muscular dystrophy [35]. However, while fat saturation reduces the lipid contribution to T_2, it does not completely eliminate the contribution of lipid with T_2 mapping. Therefore, spectroscopic relaxometry, as used in this study, has an advantage of resolving the major individual lipid resonances from water based on chemical shifts.

Fatty tissue Infiltration in Dystrophic Muscle

In contrast to ^1H_2O T_2 decreasing with age in DMD, MRI-T_2 from T_2 mapping either increased or did not change with age, depending on the muscle group. The increase in MRI-T_2 in the Sol and VL was tightly coupled with the increase in muscle lipid fraction, as shown in other studies [14,41]. This indicates that MRI signal associated with fatty tissue infiltration is the primary pathological constituent responsible for the increase in MRI-T_2 observed with older ages in DMD. Progressive lipid infiltration as assessed by MRI 3-point Dixon [17,18] and single voxel ^{1}H-MRS [19] has been associated with disease progression, age, and deterioration of clinical functional performance in DMD. However, it should be appreciated that since ^1H_2O T_2 decreases a relatively small, but measurable, amount with age, this may partially negate the increases in MRI-T_2 that occur due to lipid infiltration. For example, in this study, it is possible that MRI-T_2 could have been utilized to detect earlier changes if lipid and water T_2 contributions to overall MRI-T_2 were separated. New developments in MR acquisition and analysis have recently been proposed that enable an estimate of the water and fat contribution to MRI T_2 [14,42], and these methods may be valuable in future studies. As a result, while T_2 mapping has the potential to be influenced by both ^1H_2O T_2 and lipid infiltration, the relative contribution of each to the elevated MRI-T_2 in DMD likely varies considerably with age and disease progression. For example, ^1H_2O T_2 may be expected to be relatively more influential on MRI-T_2 at a younger age before significant lipid infiltration.

Potential of MR as outcome measures

The results of this study support muscle MRI-T_2, ^1H_2O T_2, and lipid fraction being used to monitor disease involvement in DMD, including in boys at a young age. These MR measures have recently been shown to have an excellent day-to-day reproducibility in subjects with DMD as well as across sites [21], and have the advantage of being less dependent on motivation, attention, and coordination than functional tests [9]. Furthermore, either the MRS or MRI measures enable multiple muscles to be evaluated within a relatively short amount of time. In this study, the VL

presented with earlier increases in the youngest age groups compared to the Sol, indicating the VL muscle accumulates pathology faster than the Sol. T_2 mapping and other imaging sequences, such as 3-Point Dixon [22], have an advantage of greater coverage and information about more muscle groups in a similar amount of acquisition time as single voxel ^{1}H-MRS (Table S1). With T_2 mapping there was further heterogeneity observed of different muscle groups, with the Gra, TA, and TP showing less change and the BFLH increasing at the fastest rate of the muscles analyzed in this study. Future studies would benefit from comparing the relationship of these MR measures with functional tests in a large cohort, as previously done in smaller cohorts and other dystrophies [16,20,43].

Therefore, the optimal muscle groups to target and the MR measures to acquire will likely depend on the stage of disease progression and the potential therapeutic intervention. For example, ^1H_2O T_2 may be a valuable measure for examining the effects of anti-inflammatory treatments in DMD. Other advantages of using MR to monitor disease involvement in DMD are that the measures are not limited to ambulatory patients and they have the potential to be performed in children younger than five years, particularly when motion correction strategies are implemented.

Summary

In this study ^1H_2O T_2 derived using ^{1}H-MRS and MRI-T_2 were observed to be sensitive to DMD associated pathologies, consistent with increased muscle damage and inflammation/ edema. Furthermore, muscle MRI-T_2 increased with disease progression in DMD and was strongly associated with progressive lipid infiltration as assessed by ^{1}H-MRS. Overall, this study supports that MR measures of muscle T_2 and lipid fraction may be sensitive to disease involvement and potential therapeutic interventions in DMD in all age groups, including younger boys.

Supporting Information

Table S1 Comparison of coverage in foot head direction and scan time among MR acquisitions used in this study and a 3-point Dixon scan used previously.
(DOCX)

Acknowledgments

The authors would like to thank the subjects and their families for their participation in this study.

Author Contributions

Conceived and designed the experiments: KV HLS GAW SCF WDR BJB BSR RSF MJD CRS DJL DJW. Performed the experiments: SCF RJW DJW JP DJL WDR. Analyzed the data: SCF WTT RJW MJD GAW. Contributed reagents/materials/analysis tools: SCF RJW WTT WDR DJW JP MJD GAW KV. Contributed to the writing of the manuscript: SCF RJW WTT WDR DJL DJW JP CRS MJD RSF BSR BJB ELF GIT GAW HLS KV. Analysis software development: WTT MJD GAW.

References

1. Hoffman EP, Brown RH, Kunkel LM (1987) Dystrophin: the protein product of the Duchenne muscular dystrophy locus. Cell 51: 919–928.
2. Bushby K, Finkel R, Birnkrant DJ, Case LE, Clemens PR, et al. (2010) Diagnosis and management of Duchenne muscular dystrophy, part 1: diagnosis, and pharmacological and psychosocial management. Lancet Neurol 9: 77–93.
3. Daack-Hirsch S, Holtzer C, Cunniff C (2013) Parental perspectives on the diagnostic process for Duchenne and Becker muscular dystrophy. Am J Med Genet A 161: 687–695.
4. Holtzer C, Meaney FJ, Andrews J, Ciafaloni E, Fox DJ, et al. (2011) Disparities in the diagnostic process of Duchenne and Becker muscular dystrophy. Genet Med 13: 942–947.
5. Harper SQ, Hauser MA, DelloRusso C, Duan D, Crawford RW, et al. (2002) Modular flexibility of dystrophin: implications for gene therapy of Duchenne muscular dystrophy. Nat Med 8: 253–261.
6. Kinali M, Arechavala-Gomeza V, Feng L, Cirak S, Hunt D, et al. (2009) Local restoration of dystrophin expression with the morpholino oligomer AVI-4658 in Duchenne muscular dystrophy: a single-blind, placebo-controlled, dose-escalation, proof-of-concept study. Lancet Neurol 8: 918–928.
7. Welch EM, Barton ER, Zhuo J, Tomizawa Y, Friesen WJ, et al. (2007) PTC124 targets genetic disorders caused by nonsense mutations. Nature 447: 87–91.
8. Connolly AM, Florence JM, Cradock MM, Malkus EC, Schierbecker JR, et al. (2013) Motor and cognitive assessment of infants and young boys with Duchenne Muscular Dystrophy: results from the Muscular Dystrophy Association DMD Clinical Research Network. Neuromuscul Disord 23: 529–539.
9. Hoffman EP, Connor EM (2013) Orphan drug development in muscular dystrophy: update on two large clinical trials of dystrophin rescue therapies. Discov Med 16: 233–239.
10. Henricson E, Abresch R, Han JJ, Nicorici A, Keller EG, et al. (2012) Percent-Predicted 6-Minute Walk Distance in Duchenne Muscular Dystrophy to Account for Maturational Influences. PLoS Curr.
11. Mazzone E, Martinelli D, Berardinelli A, Messina S, D'Amico A, et al. (2010) North Star Ambulatory Assessment, 6-minute walk test and timed items in ambulant boys with Duchenne muscular dystrophy. Neuromuscul Disord 20: 712–716.
12. Mazzone ES, Messina S, Vasco G, Main M, Eagle M, et al. (2009) Reliability of the North Star Ambulatory Assessment in a multicentric setting. Neuromuscul Disord 19: 458–461.
13. Mayhew AG, Cano SJ, Scott E, Eagle M, Bushby K, et al. (2013) Detecting meaningful change using the North Star Ambulatory Assessment in Duchenne muscular dystrophy. Dev Med Child Neurol 55: 1046–1052.
14. Azzabou N, Loureiro de Sousa P, Caldas E, Carlier PG (In press) Validation of a generic approach to muscle water T2 determination at 3T in fat-infiltrated skeletal muscle. J Magn Reson Imaging.
15. Friedman SD, Poliachik SL, Carter GT, Budech CB, Bird TD, et al. (2012) The magnetic resonance imaging spectrum of facioscapulohumeral muscular dystrophy. Muscle Nerve 45: 500–506.
16. Arpan I, Forbes SC, Lott DJ, Senesac CR, Daniels MJ, et al. (2012) T2 mapping provides multiple approaches for the characterization of muscle involvement in neuromuscular diseases: a cross-sectional study of lower leg muscles in 5–15-year-old boys with Duchenne muscular dystrophy. NMR in Biomed 26: 320–328.
17. Wren TAL, Bluml S, Tseng-Ong L, Gilsanz V (2008) Three-Point Technique of Fat Quantification of Muscle Tissue as a Marker of Disease Progression in Duchenne Muscular Dystrophy: Preliminary Study. Am J Roentgenol 190: W8–12.
18. Wokke BH, van den Bergen JC, Versluis MJ, Niks EH, Milles J, et al. (2014) Quantitative MRI and strength measurements in the assessment of muscle quality in Duchenne muscular dystrophy. Neuromuscul Disord 24: 409–416.
19. Torriani M, Townsend E, Thomas B, Bredella M, Ghomi R, et al. (2012) Lower leg muscle involvement in Duchenne muscular dystrophy: an MR imaging and spectroscopy study. Skeletal Radiol 41: 437–445.
20. Lott DJ, Forbes SC, Mathur S, Germain SA, Senesac CR, et al. (2014) Assessment of Intramuscular Lipid and Metabolites of the Lower Leg using Magnetic Resonance Spectroscopy in boys with Duchenne muscular dystrophy. Neuromuscul Disord 24: 574–582.
21. Forbes SC, Walter GA, Rooney WD, Wang D-J, DeVos S, et al. (2013) Skeletal Muscles of Ambulant Children with Duchenne Muscular Dystrophy: Validation of Multicenter Study of Evaluation with MR Imaging and MR Spectroscopy. Radiology 269: 198–207.
22. Triplett WT, Baligand C, Forbes SC, Willcocks RJ, Lott DJ, et al. (2014) Chemical shift-based MRI to measure fat fractions in dystrophic skeletal muscle. Magn Reson Med 72: 8–19.
23. Mathur S, Vohra RS, Germain SA, Forbes S, Bryant ND, et al. (2011) Changes in muscle T2 and tissue damage after downhill running in mdx Mice. Muscle Nerve 43: 878–886.
24. Arpan I, Willcocks RJ, Forbes SC, Finkel RS, Lott DJ, et al. (In press) Examination of Effects of Corticosteroids on Skeletal Muscles of Boys with DMD using MRI and MRS. Neurology.
25. Frahm J BH, Gyngell ML, Merboldt KD, Hänicke W, Sauter R. (1989) Localized high-resolution proton NMR spectroscopy using stimulated echoes: initial applications to human brain in vivo. Magn Reson Med 9: 79–93.
26. Bruhn H FJ, Gyngell ML, Merboldt KD, Hänicke W, Sauter R. (1991) Localized proton NMR spectroscopy using stimulated echoes: applications to human skeletal muscle in vivo. Magn Reson Med 17: 82–94.
27. Maier CF, Tan SG, Hariharan H, Potter HG (2003) T2 quantitation of articular cartilage at 1.5 T. J Magn Reson Imaging 17: 358–364.

28. Gold GE, Han E, Stainsby J, Wright G, Brittain J, et al. (2004) Musculoskeletal MRI at 3.0 T: Relaxation Times and Image Contrast. AJR Am J Roentgenol 183 343–351.
29. Krssak M, Roden M, Mlynarik V, Meyerspeer M, Moser E (2004) 1H NMR relaxation times of skeletal muscle metabolites at 3 T. Magnetic Resonance Materials in Physics, Biology and Medicine 16: 155–159.
30. Elliott MA, Walter GA, Swift A, Vandenborne K, Schotland JC, et al. (1999) Spectral quantitation by principal component analysis using complex singular value decomposition. Magn Reson Imaging 41: 450–455.
31. Forbes SC, Lott DJ, Finkel RS, Senesac C, Byrne BJ, et al. (2012) MRI/MRS evaluation of a female carrier of Duchenne muscular dystrophy. Neuromuscul Disord 22, Supplement 2: S111–S121.
32. Petrof BJ (2006) Molecular pathophysiology of myofiber injury in deficiencies of the dystrophin-glycoprotein complex. Am J Phys Med Rehabil 81: S162–174.
33. Kobayashi YM, Rader EP, Crawford RW, Iyengar NK, Thedens DR, et al. (2008) Sarcolemma-localized nNOS is required to maintain activity after mild exercise. Nature 27: 511–515.
34. Weber MA, Nagel AM, Wolf MB, Jurkat-Rott K, Kauczor HU, et al. (2012) Permanent muscular sodium overload and persistent muscle edema in Duchenne muscular dystrophy: a possible contributor of progressive muscle degeneration. J Neurol 259: 2385–2392.
35. Frisullo G, Frusciante R, Nociti V, Tasca G, Renna R, et al. (2011) CD8+ T Cells in Facioscapulohumeral Muscular Dystrophy Patients with Inflammatory Features at Muscle MRI. J Clin Immunol 31: 155–166.
36. Foley JM, Yayaraman RC, Prior BM, Pivarnik JM, Meyer RA (1999) MR measurements of muscle damage and adaptation after eccentric exercise. J Appl Physiol 87: 2311–2318.
37. Rooney WD, Forbes SC, Triplett W, Wang D-J, Pollaro J, et al. (2013) Soleus Muscle Water T2 Values in Duchenne Muscular Dystrophy: Associations with Age and Corticosteroid Treatment Proc Intl Soc Mag Reson Med 21: 0689.
38. Loganathan R, Bilgen M, Al-Hafez B, Smirnova IR (2005) Characterization of alterations in diabetic myocardial tissue using high resolution MRI. Int J Cardiovasc Imaging 22: 81–90.
39. Marden FA, Connolly AM, Siegel MJ, Rubin DA (2005) Compositional analysis of muscle in boys with Duchenne muscular dystrophy using MR imaging. Skeletal Radiol 34: 140–148.
40. Friedman SD, Poliachik SL, Otto RK, Carter GT, Budech CB, et al. (2014) Longitudinal Features of Stir Bright Signal in FSHD. Muscle Nerve 49: 257–260.
41. Gloor M, Fasler S, Fischmann A, Haas T, Bieri O, et al. (2011) Quantification of fat infiltration in oculopharyngeal muscular dystrophy: Comparison of three MR imaging methods. J Magn Reson Imaging 33: 203–210.
42. Carlier PG (2014) Global T2 versus water T2 in NMR imaging of fatty infiltrated muscles: Different methodology, different information and different implications. Neuromuscul Disord 24: 390–392.
43. Willis TA, Hollingsworth KG, Coombs A, Sveen M-L, Andersen Sr, et al. (2013) Quantitative Muscle MRI as an Assessment Tool for Monitoring Disease Progression in LGMD2I: A Multicentre Longitudinal Study. PLoS One 8: e70993.
